# Minimally Invasive Video-Assisted versus Minimally Invasive Nonendoscopic Thyroidectomy

**DOI:** 10.1155/2014/450170

**Published:** 2014-04-08

**Authors:** Zdeněk Fík, Jaromír Astl, Michal Zábrodský, Petr Lukeš, Ilja Merunka, Jan Betka, Martin Chovanec

**Affiliations:** ^1^Department of Otorhinolaryngology, Head and Neck Surgery, First Faculty of Medicine, Charles University in Prague and University Hospital Motol, V Úvalu 84, 150 06 Prague 5, Czech Republic; ^2^Institute of Anatomy, First Faculty of Medicine, Charles University in Prague, U Nemocnice 3, 128 00 Prague 2, Czech Republic; ^3^Department of Otorhinolaryngology, Head and Neck Surgery, Third Faculty of Medicine, Charles University Prague, Military Hospital, U Vojenské Nemocnice 1200, 169 00 Prague 6, Czech Republic; ^4^Department of Electromagnetic Field, Faculty of Electrical Engineering, Czech Technical University in Prague, Technická 2, 166 27 Prague 6, Czech Republic

## Abstract

Minimally invasive video-assisted thyroidectomy (MIVAT) and minimally invasive nonendoscopic thyroidectomy (MINET) represent well accepted and reproducible techniques developed with the main goal to improve cosmetic outcome, accelerate healing, and increase patient's comfort following thyroid surgery. Between 2007 and 2011, a prospective nonrandomized study of patients undergoing minimally invasive thyroid surgery was performed to compare advantages and disadvantages of the two different techniques. There were no significant differences in the length of incision to perform surgical procedures. Mean duration of hemithyroidectomy was comparable in both groups, but it was more time consuming to perform total thyroidectomy by MIVAT. There were more patients undergoing MIVAT procedures without active drainage in the postoperative course and we also could see a trend for less pain in the same group. This was paralleled by statistically significant decreased administration of both opiates and nonopiate analgesics. We encountered two cases of recurrent laryngeal nerve palsies in the MIVAT group only. MIVAT and MINET represent safe and feasible alternative to conventional thyroid surgery in selected cases and this prospective study has shown minimal differences between these two techniques.

## 1. Introduction

Thyroid surgery belongs among the most common procedures on the neck. The first attempts to treat diseases of the thyroid gland surgically are dated back to antiquity [1]. However modern concept of the treatment had been developed at the beginning of the 19th century and it is still relevant till these days [2].

The goal for the surgeon is to remove the whole gland or its specific part, preserving inferior and superior laryngeal nerves, parathyroid glands, while achieving safe hemostasis mainly by ligating superior and inferior thyroid arteries [3].

The most common approach to the gland remains the same for decades—standard Kocher incision on the neck, two centimeters above the jugulum [4]. At the end of 20th century many other approaches were studied and gradually introduced into the practice. Pioneer work of Gagner et al described cervical endoscopic parathyroidectomy [5]. Subsequently, surgeon Miccoli with co-workers applied endoscopic technique to the thyroid gland [6]. In his future work he has elaborated methodics for minimally invasive video-assisted thyroidectomy (MIVAT), performed via small incision on the neck (<3 cm), using endoscope and special instruments for dissection [7].

Minimally invasive nonendoscopic thyroidectomy (MINET, MIT) is technically less demanding, also based on experiences from parathyroid surgery [8]. First report of MINET was published by Ferzli et al., who explored the neck trough 2.5 cm cervical incision, using only headlight to improve visualization [9]. Currently the definition of the MINET is a thyroidectomy, performed without endoscope, using incision of less than 3.5 cm [10]. Use of tools, magnifying the operation field, such as microscope or loupes, is worthwhile but not necessary [11, 12].

Common goal of minimally invasive approaches is better cosmetic results, decreased postoperative pain, and shortening of inpatient period, without an increase in the postoperative complication rates [13]. To achieve these demands it is useful to abide strict indication criteria. For MIVAT, these were stated by Miccoli et al. as thyroid nodules smaller than 30 mm in their largest diameter, thyroid glands with a volume less than 25 mL, absence of the thyroid gland fixation (thyroiditis, history of neck irradiation or previous neck surgery and extrathyroid cancer spread), follicular tumor or “low risk” papillary carcinoma, and RET gene mutation carriers-elective thyroidectomy [7].

There are not so detailed criteria for MINET, but principally the surgeon's restrictions are still the volume of the gland and the extent of its fixation to the surrounding structures; however present indication criteria are more benevolent [14].

Different approach to the thyroid gland was explored in Japan, where Ikeda et al. developed endoscopic approach via axillary incision [15]. Since then, many modifications of these so-called extracervical approaches were developed (see review by Touzopoulos et al. [13]). Development in robotic surgery facilitated introduction of novel approaches, such as transoral video-assisted thyroidectomy (TOVAT) or retroauricular approach (RA) [16, 17].

The aim of this study was to perform prospective analysis of MINET and MIVAT technique.

## 2. Material and Methods

In our prospective study we compared thyroid surgeries, performed with MIVAT and MINET technique. The basic inclusion criteria were the preoperative ultrasonography (USG) dimensions of the thyroid gland, with volumetric limit of 55 mL of one thyroid lobe and the maximum size of the thyroid nodule, not exceeding 35 mm. All thyroid volumes were adjusted according to formula: volume = *f* × (*A* × *B* × *C*) × 10^−3^, in which letters *A*, *B*, and *C* represent the maximal sizes in the two mutually perpendicular plains, measured on USG, and *f* is the correction factor for ellipsoid models, which corresponds to *π*/6 = 0.524 [18].

The exclusion criteria were significant retrosternal thyroid extension, suspicion for regional metastatic spread of the thyroid cancer, laboratory confirmed chronic thyroiditis, Hashimoto's disease (positivity for anti-TPO and/or anti-TG), and pathologic gland fixation to the surrounding tissues.

Surgeries were performed by five surgeons during the period 2007–2011. Two expert surgeons (more than 1000 thyroid surgeries) performed 49 MINET and 21 MIVAT operations. Two moderately experienced surgeons (more than 200 thyroid surgeries) performed 22 MINET and 38 MIVAT operations. One beginning surgeon performed 1 MIVAT under the supervision of above-mentioned surgeons and no MINET operation. Each surgical procedure was performed by team of three surgeons, one performing the procedure and two assisting during thyroidectomy. Surgical loupes were employed during MINET procedures. Endoscope and special instruments for dissection (KARL STORZ GmbH & Co. KG, Tuttlingen, Germany) were employed for MIVAT.

All patients, who were potential candidates for minimally invasive thyroid surgery, were informed about the character of conventional and both minimally invasive approaches and given possibility to participate in the study, submitting informed consent.

There were 62 patients undergoing MIVAT procedures; however in two cases MIVAT surgery had to be converted to the conventional technique. In the first case, extracapsular spread of the tumor into the trachea and esophagus required more extensive approach. In the second case, the preoperative ultrasonography underestimated the size of gland and its pathology; thus MIVAT procedure was abandoned for the safety reason. These two cases were not included in the study and overall 60 patients were enrolled. In the MIVAT group of procedures there were 40 total thyroidectomies (MIVAT-TTE), 18 hemithyroidectomies (MIVAT-HTE), and 2 isthmectomies. Isthmectomies as partial procedures were analyzed together with hemithyroidectomies. Overall 71 patients were undergoing MINET. Of these 41 total thyroidectomies (MINET-TTE) and 30 hemithyroidectomies (MINET-HTE) were performed (Table 1).

The analyzed parameters comprised length of incision, duration of the surgery, postoperative blood loss, postoperative pain and its treatment, inpatient period, and postoperative complications.

The length of incision was measured with ruler, following the incision at the beginning of procedure. The duration of surgery was defined from the incision to the wound closure.

Postoperative blood loss was deduced from the vacuum drain bottle till its removal (the bottle was removed when the blood loss decreased below 20 mL/24 hours) [19]. Eventual application of the active drainage system was evaluated too.

Visual analogue scale (VAS) was employed to evaluate the postoperative pain (VAS 0–10; 0 corresponding to no pain, 10 corresponding to the most severe pain, which is patient able to imagine) [20]. The pain was measured at the end of the 1st, 6th, and 12th postoperative hour. Together with the pain evaluation the consumption of analgesics (opiate and nonopiate) was matter of observation. Nonopiate analgesics were administered if postoperative pain exceeded VAS 2. In case the VAS ≥ 5 opiate analgesics (pethidine) were administered.

Serum levels of calcium and inorganic phosphates were measured during first and second postoperative day. If the hypocalcaemia was present (serum Ca < 2 mmol/L), calcium level was measured every day till its normalization. In case of prolonged hypocalcaemia or Ca < 1.8 mmol/L we indicated measurement of serum parathormone (PTH) to exclude hypoparathyreosis.

Pre- and postoperative evaluation of inferior laryngeal nerve function were evaluated with video-laryngoscopy together with video-stroboscopy and video-kymography [21].

Analgesics consumption and suction drainage employment were statistically correlated using Chi-square test. For the rest of the data, two-sample* t*-test was used. Difference of *P* < 0.05 was accepted as significant and difference of *P* < 0.01 as highly significant.

## 3. Results

There were 6 males and 54 females (40 ± 14 years) undergoing MIVAT. In the group of patients undergoing MINET there were 6 males and 65 females (37 ± 14 years) (Table 1). Volume of thyroid lobes and size of nodules were comparable in both studied groups (Table 2).

The most prevailing diagnosis managed by MIVAT was benign follicular neoplasm (*n* = 36) followed by well differentiated carcinomas (*n* = 17) and toxic goiters (*n* = 7). Similarly in the MINET group the most common type of pathology treated was benign follicular neoplasm (*n* = 42), followed by well differentiated carcinomas (*n* = 18) and toxic goiters (*n* = 10). We performed also one elective MINET-TTE due to hereditary burden of medullary thyroid carcinoma-positivity of RET proto-oncogene (Table 3).

There were no significant differences in the length of incision to perform either hemithyroidectomy (19 ± 6 mm in MIVAT versus 23 ± 5 mm in MINET) or total thyroidectomy (25 ± 5 mm in MIVAT versus 23 ± 5 mm in MINET) (Table 4).

Mean duration of hemithyroidectomy was comparable in both groups (77 ± 24 min in MIVAT versus 71 ± 15 min in MINET). It was more time-consuming to perform total thyroidectomy by MIVAT than MINET technique (108 ± 33 min in MIVAT versus 96 ± 18 min in MINET; *P* = 0.05) (Table 4).

Among the patients undergoing hemithyroidectomy there were 5 patients without active drainage in the MIVAT-HTE group. Surprisingly the average blood loss, deduced from bottle of active drainage, was slightly lower in patients from the MINET-HTE group (42 ± 39 mL in MIVAT versus 34 ± 19 mL in MINET). Overall, 3 patients following MIVAT-TTE and 2 patients following MINET-TTE were recovering without active drainage. There were no significant differences in the blood loss across both groups undergoing total thyroidectomy (61 ± 32 mL in MIVAT versus 63 ± 25 mL in MINET) (Table 4).

There was trend for lower postoperative pain in the MIVAT group assessed by VAS scores at 1st, 6th, and 12th postoperative hour in both patients undergoing hemithyroidectomy and total thyroidectomy (Table 5). This trend was paralleled by decreased opiates and total analgesics consumption. All patients in our series of the minimally invasive thyroid surgery demanded opiate analgesics only during the first postoperative day.

Following MIVAT-TTE fourteen (36%) patients were not administered opiates compared to seven patients (19%) in the MINET-TTE group. There was highly statistically significant difference in the consumption of nonopiate analgesics as these were given to three patients (8%) in the MIVAT-TTE group compared to fourteen patients (34%) in the MINET-TTE group (*P* < 0.01). Thirty-six patients (93%) in the MIVAT-TTE group were not administered any analgesic since the first postoperative day, compared to twenty-six patients (71%) in the MINET-TTE group (*P* = 0.01). Finally thirteen patients (33%) undergoing MIVAT-TTE did not need any analgesics postoperatively compared to six patients (15%) in the MINET-TTE group (Table 5).

Among patients undergoing hemithyroidectomy seven patients (35%) from the MIVAT group and seven patients (25%) from the MINET group were not administered opiates. In both groups 5 patients (25% in the MIVAT group and 17% in the MINET group) did not need any analgesics following the surgery. Following MIVAT-HTE 17 patients (85%) did not require any analgesic and 24 patients (86%) undergoing MINET-HTE since the first postoperative day (Table 5).

Concerning the postoperative complications we encountered two cases of recurrent laryngeal nerve palsies in the MIVAT group compared to no palsy in the MINET group. One case of palsy following hemithyroidectomy was transient. It improved to normal function three months following surgery. The second palsy as a consequence of direct recurrent laryngeal nerve injury was managed by immediate neurorrhaphy. Despite this procedure the vocal fold palsy is permanent; however good quality of voice was achieved paralleled by acquisition of optimal vibratory characteristics on video-kymography.

There were 5 transient symptomatic hypocalcaemias following MIVAT-TTE compared to 7 hypocalcaemias following MINET-TTE. The similar ratio was observed in the case of asymptomatic hypocalcaemias (10 in MIVAT-TTE versus 15 in MINET-TTE). No difference was observed in the average values of the serum calcaemia. All patients with postoperative hypocalcaemia achieved satisfactory improvement with transient substitution. Postoperative hypoparathyreosis was not observed in both groups studied.

We encountered postoperative bleeding in 2 patients undergoing MIVAT-TTE and 1 patient following MINET-HTE. All cases of bleeding occurred in the operating theater just before extubation and were indicated to immediate revision. One seroma and two wound infections were observed in the MIVAT group in cases managed without active drainage. As such all were managed conservatively.

The inpatient period in patients undergoing hemithyroidectomy was slightly shorter in the MIVAT group (2.8 ± 0.8 in MIVAT-HTE versus 3 ± 0.7 in MINET-HTE; 2.8 ± 0.8 in MIVAT-TTE versus 3 ± 0.7 in MINET-TTE).

## 4. Discussion

Our previous work prospectively comparing conventional and minimally invasive video-assisted thyroidectomy proved both techniques to be equally effective, with some advantages in favor of minimally invasive surgery [22]. Actual analysis prospectively compared thyroid surgeries, performed with both minimally invasive nonendoscopic and minimally invasive video-assisted approach. Studied groups were comparable for demographic parameters of patients, pathologies treated including the size of nodules and volume of the gland resected and experience of surgeon. Our results show clear benefits of both techniques employed.

The main statistically significant difference emerging from this study was analgesics consumption. Decreased nonopiate analgesics administration was observed in the MIVAT group. This could be attributed to the necessity of significant retraction during MINET surgery as compared to the MIVAT procedures. However Perigli et al. in their study did not find any difference in the postoperative pain, as well as in cosmetic result and hypoparathyroidism in the groups of MIVAT and MINET surgeries [23]. Furthermore no histological changes in structure of the scar were found when comparing specimens of different retraction forces [24].

Despite good reproducibility of both minimally invasive techniques it is difficult to find works, comparing these two methods. Ferzli et al. described their experience with minimally invasive nonendoscopic thyroid surgery and discussed its advantages and disadvantages compared to completely endoscopic, as well as video-assisted surgery [9].

Regarding MIVAT, after five patients operated, Ferzli et al. found this approach more time consuming than nonendoscopic method and more demanding for technique skills. Limited amount of treated patients does not offer representative data for clear conclusion.

Presented data proved comparable surgical time when performing MIVAT and MINET hemithyroidectomies. On the other hand, MIVAT complete thyroidectomies were more time consuming than MINET, despite that the results were not significant. It can be estimated that video-endoscopic procedures should take more time due to the setup of the video-screen during surgery, but the operation time should decrease with growing surgeon's skills. For example, del Rio et al. discussed a learning curve, which should reduce the time of surgery with more than 20 video-assisted thyroidectomies [25]. Comparing the length of surgeries to the results of published works it is necessary to understand the structure of the surgical team. Presented data show results of surgeries performed by the team of three surgeons when the first assistant participates during the tissue dissection and work with video-endoscope, while the second assistant is fully involved in wound retraction. Thus teams composed of four surgeons have clear benefit of one assistant being fully involved in the work with video-endoscope without the need for frequent extraction of endoscope from the wound as the assistance during tissue dissection can be performed by another active surgeon.

There was no significant difference in the length of incision in both groups. Furthermore the upper limit of incision length was defined as 3 cm [26]. On the other hand, the length of incision could not be considered as the only criteria of the minimally invasive approach [23]. Henry et al. regarded minimally invasive thyroid surgery as the procedure, using lesser extent of dissection space as compared to conventional technique [27]. Furthermore some works question the importance of the small scar for patient's satisfaction after thyroid surgery [28].

There is a prediction that employment of minimally invasive approach should decrease postoperative blood loss. Our previous study analyzing results of MIVAT and conventional thyroidectomy confirmed such data [22]. Comparing both minimally invasive techniques, postoperative blood loss did not show any significant difference between the two groups. Furthermore the amount of patients who did not require active drainage was equal. However there are some problems in quantifying blood loss following operation. Foremost the starting negative pressure in the bottle is probably not constant and its subsequent decrease depends also on the volume of the dissected space. Furthermore, correlation with anticoagulants consumption and preoperative levels of anticoagulation parameters should be considered. The question of active drainage employment assessment remains controversial, because the decision also depends on the experience and self-confidence of the surgeon. All cases of postoperative bleeding (two following MIVAT and one following MINET) occurred still in the surgical theatre following wound closure and were managed swiftly with wound revision.

In all patients, undergoing total thyroidectomy because of the thyroid cancer, complete removal of the gland (athyreosis) was achieved being confirmed with laboratory examination (serum thyreoglobulin level) and scintigraphy. There was one case of extracapsular spread with tracheal invasion identified during MIVAT procedure; thus surgeon was forced to convert to the conventional technique. In other cases of WDTC there were no difficulties with the gland removal either during MIVAT or MINET. MIVAT is considered as safe procedure for small WDTC (T1 and T2 tumors) [29, 30] but should be avoided in anaplastic thyroid cancer or in the neck node positive cases [31]. On the other hand, video-assisted neck dissection is recently discussed topic [32] and minimally invasive video assisted thyroidectomy with the central neck dissection is current* state of the art* [33].

One case of the unilateral inferior laryngeal nerve injury was encountered in the MIVAT performed by an expert thyroid surgeon. Immediate neurorrhaphy was performed. Absence of vocal fold mobility was observed during postoperative follow-up; however video-kymography and video-stroboscopy have shown acquisition of consecutive synchronization of the mucosal wave during laryngeal adaptation to the changes in innervation. No signs of atrophy, as well as impairment of the vocal fold position, were observed. The patient successfully rehabilitated the voice and avoided corrective surgery (augmentation, vocal fold medialisation) [34].

One case of transient unilateral inferior laryngeal nerve palsy following MIVAT-HTE had identical initial video-kymography finding as permanent palsy. Full vocal fold mobility and video-kymographic findings verified improvement of function* ad integrum* after few months [34].

We defined our inclusion criteria for minimally invasive surgery as thyroid volume ≤ 55 mL and the largest size of the nodule ≤ 35 mm; thus we did not meet the mentioned Miccoli's criteria [7] in eight cases. MINET-HTE/TTE was performed also on thyroids with nodule sizes in the range 36–50 mm. Nodule sizes 45 mm and 49 mm were present at the patients, undergoing MIVAT-TTE. All thyroids with nodules > 35 mm were amenable to minimally invasive approach as nodules were elliptic with the smaller dimension < 35 mm and total lobe volume < 50 mL. Thyroids with such volumes were advocated by Ruggieri et al. as feasible for MIVAT surgery [35]. Some works advocates minimally invasive techniques for thyroid glands, exceeding micolli's crietria mentioned above [36–38].

There are not so detailed criteria for MINET, but principally the surgeon's restrictions are still the volume of the gland and the extent of its fixation to the surrounding tissues [14]. Works describing lateral mini-incision technique (<2.5 cm) for benign thyroid nodules confirm such statement [39, 40]. Therefore in our opinion criteria adopted for MIVAT are true for MINET surgery as well. With such inclusion criteria only 10% of patients with thyroid disease represent potential candidates for minimally invasive surgery [41].

One video-assisted and one nonendoscopic thyroidectomy were performed in the setting of Hashimoto thyroiditis. However this disease belongs to a list of contraindications [7]. Both surgeries went uneventful with complete removal of the gland. On the other hand, the length of MIVAT was 160 minutes, due to hypervascularization of the gland. Currently, some works consider Hashimoto thyroiditis as a relative contraindication for such approach [25, 36, 42, 43].

Previous radiotherapy on the neck region is regarded as contraindication for minimally invasive approaches [7, 25, 44]. There was one patient in our study, who underwent radiotherapy for hematological malignancy during childhood (neck was in the irradiated field). With regard to soft neck during palpation with unfixed thyroid gland, we performed MIVAT surgery without difficulties.

We can conclude that Miccoli's criteria [7] are not strictly dogmatic, but surgeon has to create all conditions to perform safe procedure.

## 5. Conclusion

MIVAT and MINET represent safe procedures, which can offer the patient a benefit of better cosmetic outcome and postoperative recovery, without increasing amount of complication. This prospective study has shown minimal differences between these two minimally invasive techniques, apart from analgesics administration in behalf of video-assisted approach. Hand in hand with increasing knowledge, skills, and improvement of material equipment, it is possible to review possible indications and offer minimally invasive techniques to a larger group of patients; however motivation to reach better cosmetic results is controversial.

## Figures and Tables

**Table 1:** Patient characteristics.

	MIVAT	MINET
*N*	60	71
TTE	40	41
HTE	20*	30
Age (years)	40 ± 14 (17–75)	37 ± 14 (10–68)
Gender (males/females)	6/54	6/66

MIVAT: minimally invasive video-assisted thyroidectomy; MINET: minimally invasive nonendoscopic thyroidectomy; TTE: total thyroidectomy; HTE: hemithyroidectomy; *2 cases of isthmectomies were analyzed together with hemithyroidectomies.

**Table 2:** Volume of thyroid lobe and nodule size as assessed on preoperative ultrasonography.

	MIVAT	MINET
Nodule size	20 ± 11 (3–49) mm	21 ± 10 (3–50) mm
Volume of the lobe	12 ± 7 (3–56) mL	12 ± 7 (3–35) mL

MIVAT: minimally invasive video-assisted thyroidectomy; MINET: minimally invasive nonendoscopic thyroidectomy.

**Table 3:** Treated thyroid gland pathologies.

Pathology treated	MIVAT	MINET
Benign follicular neoplasm	36	42
WDTC	17	18
T1	13	12
T2	1	4
T3	3	2
Thyreotoxicosis	7	10
GBD	5	6
Toxic adenoma	1	
Amiodaron induced toxicosis	1	
Nootropil induced toxicosis		1
Plummer's disease		3

MIVAT: minimally invasive video-assisted thyroidectomy, MINET: minimally invasive non-endoscopic thyroidectomy, WDTC: well-differentiated thyroid cancer, T1–T3: T-staging according 7th edition of TNM classification of malignant tumors, GBD: Graves-Basedow disease.

**Table 4:** Size of incision, duration of surgery, and postoperative blood loss.

	MIVAT-HTE	MINET-HTE	*P*	MIVAT-TTE	MINET-TTE	*P*
Length of incision	19 ± 6 (11–30) mm	23 ± 5 (15–30) mm	0.06	25 ± 5 (15–30) mm	26 ± 5 (15–30) mm	0.30
Duration of surgery	77 ± 24 (47–150) min	71 ± 15 (44–100) min	0.98	108 ± 33 (60–240) min	96 ± 18 (62–130) min	0.05
Blood loss*	42 ± 39 (10–170) mL	34 ± 19 (10–70) mL	0.49	61 ± 32 (14–120) mL	63 ± 25 (20–140) mL	0.83

MIVAT: minimally invasive video-assisted thyroidectomy; MINET: minimally invasive nonendoscopic thyroidectomy; *measured from the bottle of the active drainage.

**Table 5:** Postoperative pain and analgesics consumption.

	MIVAT-HTE	MINET-HTE	*P*	MIVAT-TTE	MINET-TTE	*P*
Postoperative pain						
VAS^1 h postoperatively^	1.7 ± 1.4 (0–8)	1.8 ± 1.7 (0–6)	0.96	1.9 ± 1.5 (0–5)	2.4 ± 2 (0–6)	0.18
VAS^6 h postoperatively^	1.5 ± 1.5 (0–5)	1.7 ± 1.3 (0–5)	0.57	1.2 ± 1.1 (0–5)	1.2 ± 1.4 (0–5)	0.66
VAS^12 h postoperatively^	0.3 ± 0.6 (0–2)	1.5 ± 0.8 (0–5)	0.49	0.6 ± 1 (0–4)	1.1 ± 1.6 (0–5)	0.07
Analgesics consumption						
Opiates*	13	23	0.37	26	34	0.07
Nonopiates**	3	6	0.65	3	14	<0.01
Without analgesics postoperatively	5	5	0.47	13	6	0.06
Without analgesics since 1st postoperative day	17	24	0.65	36	26	0.01

MIVAT: minimally invasive video-assisted thyroidectomy; MINET: minimally invasive nonendoscopic thyroidectomy; VAS: 10 grade visual analogue scale; *opiates administered at the day of surgery; **nonopiates administered since the surgery.
